# Candidates for Repurposing as Anti-Virulence Agents Based on the Structural Profile Analysis of Microbial Collagenase Inhibitors

**DOI:** 10.3390/pharmaceutics14010062

**Published:** 2021-12-28

**Authors:** Georgiana Nitulescu, George Mihai Nitulescu, Anca Zanfirescu, Dragos Paul Mihai, Daniela Gradinaru

**Affiliations:** Faculty of Pharmacy, “Carol Davila” University of Medicine and Pharmacy, Traian Vuia 6, 020956 Bucharest, Romania; georgiana.nitulescu@umfcd.ro (G.N.); anca.zanfirescu@umfcd.ro (A.Z.); dragos_mihai@drd.umfcd.ro (D.P.M.); daniela.gradinaru@umfcd.ro (D.G.)

**Keywords:** metalloproteinase inhibitors, Bemis-Murcko skeletons, molecular docking, repurposing, antimicrobial resistance, antibiotics, benzthiazide, entacapone, lodoxamide

## Abstract

The pharmacological inhibition of the bacterial collagenases (BC) enzymes is considered a promising strategy to block the virulence of the bacteria without targeting the selection mechanism leading to drug resistance. The chemical structures of the *Clostridium perfringens* collagenase A (ColA) inhibitors were analyzed using Bemis-Murcko skeletons, Murcko frameworks, the type of plain rings, and docking studies. The inhibitors were classified based on their structural architecture and various scoring methods were implemented to predict the probability of new compounds to inhibit ColA and other BC. The analyses indicated that all compounds contain at least one aromatic ring, which is often a nitrobenzene fragment. 2-Nitrobenzene based compounds are, on average, more potent BC inhibitors compared to those derived from 4-nitrobenzene. The molecular descriptors MDEO-11, AATS0s, ASP-0, and MAXDN were determined as filters to identify new BC inhibitors and highlighted the necessity for a compound to contain at least three primary oxygen atoms. The DrugBank database was virtually screened using the developed methods. A total of 100 compounds were identified as potential BC inhibitors, of which, 10 are human approved drugs. Benzthiazide, entacapone, and lodoxamide were chosen as the best candidates for in vitro testing based on their pharmaco-toxicological profile.

## 1. Introduction

Antibiotic resistance is a major health issue and is spreading dangerously throughout the world, threatening our ability to treat common infectious diseases. Moreover, pathogens are resistant to more than one class of antimicrobial agents. *Pseudomonas aeruginosa*, *Acinetobacter baumannii*, *Escherichia coli*, and *Klebsiella pneumoniae* bearing extended-spectrum β-lactamases (ESBL), vancomycin-resistant enterococci (VRE), methicillin-resistant *Staphylococcus aureus*, *Clostridium difficile* are among these multidrug resistant (MDR) problematic species, causing great morbidity and mortality, particularly in hospitals and other healthcare institutions [1].

Developing new antibiotics has become undoubtedly a race against time, as the emergence of MDR pathogens is outpacing the development of these therapeutic agents [2]. Understanding bacterial pathogenesis and resistance mechanisms, such as antibiotic efflux, antibiotic inactivation, biofilm formation, and target modification, as well as intercellular communication has revealed many potential strategies to develop novel drugs to treat bacteria-mediated diseases.

One of these new strategies include developing anti-virulence therapies—virulence being defined as the relative capacity of a microbe to cause damage to a host [3]. As opposed to traditional antibiotics which that bacterial growth pathways, anti-virulence offers an alternative approach that focuses on interfering with bacterial virulence factors (e.g., exotoxins, biofilm, secreted enzymes), thus interrupting the infectious process rather than bacterial growth [4,5,6,7].

Various research groups reported the existence of a promising number of anti-virulence strategies: inhibition of pore-forming toxins that kill host cells, thereby combatting immune responses and liberating nutrients from the host [8]; inhibition of microbial adhesion and colonization (e.g., inhibition of pili formation by binding the PapD protein, a conserved chaperone for pilus assembly in uropathogenic *E. coli*) [9]; inhibition of the secretion of cell surface proteins (e.g., sortases) [7]; inhibition of regulatory bacterial function (e.g., targeting quorum sensing, a complex regulatory network that governs the expression of a series of bacterial virulence factors in response to cell density or environment changes) [10]; inhibition of the bacterial cell wall resistance to the innate immunity (e.g., inhibition of heptose biosynthesis, heptose being the structural element within the conserved lipopolysaccharide core—the major component of the outer membrane of Gram-negative bacteria and a major determinant of their pathogenicity) [11].

Drugs targeting anti-virulence factors are intended to be used for the treatment of bacterial infections with multi-resistant pathogens, as monotherapy or associated with classical antibacterial substances. They are also expected to possess several advantages over the latter, such as reduced selection pressure and minimal perturbation of the healthy microbiota. Some have already been approved by the US Food and Drug Administration and/or the European Medicines Agency for bacterial toxin-mediated diseases (e.g., raxibacumab, obiltoxaximab), while others targeting antibiotic-resistant bacteria have entered clinical trials (e.g., AR-301, MEDI4893) [12].

As mentioned above, inhibition of bacterial adhesion and colonization represents a promising anti-virulence strategy. We focus on bacterial collagenases (BC) as important virulence factors involved in the growth and proliferation of pathogenic bacteria on their hosts. These proteolytic enzymes possess broad substrate specificity—they degrade both water insoluble and water soluble collagens in their triple helical regions at X-Gly bonds [13]. Due to their ability to degrade specific cell-membrane or extracellular-matrix components, they play key roles in host colonization. They contribute to the spread of the pathogen into the host tissues, ensure the influx of proper nutrients for survival and growth, e.g., amino acids into the bacterial cells, and facilitate toxins’ diffusion [14,15].

Virulence of pathogenic bacteria was associated mostly with collagenases belonging to the M9 family or with bacterial collagenolytic proteases [16], members of the U32 family in the MEROPS database [17]. The MEROPS M9 peptidase family comprise metalloproteases with a conserved zinc-binding motif, either (HEY/FTH) or (HEYT/VH), which functions as a catalytic domain [18]. They act as true bacterial collagenases, cleaving helical regions of fibrillar collagen molecules under physiological conditions [19]. They belong either to the M9A or to M9B subfamilies, based on differences in their amino acid sequence and catalytic function. Class I enzymes cannot digest collagen, class II enzymes cannot hydrolyze casein but are able to digest collagen, while class III enzymes are able to digest caseins, gelatin, and collagen [19]. Bacterial collagenolytic pathogenic bacteria and their collagenases are presented in Table 1.

Collagenases are reported to be important in the pathogenic process of other bacteria, such as *Pseudomonas aeruginosa*, *Proteus mirabilis*, *B. anthracis*, and *Leptospira* sp., although their specificity needs further investigation [32,33,34,35]. Thus, targeting collagenases could provide a suitable strategy for developing new anti-virulence agents [36,37,38]. We focused on identifying inhibitors that could directly inactivate this specific virulence factor by using drug repurposing—taking already approved drugs and using them outside of their original designated medical indications. The computational drug repurposing strategy was shown to greatly reduce time and costs generally associated with standard drug discovery processes and to prevent any events related to unpredicted toxicity of a new active substance [39,40,41,42].

Using molecular docking and analyses of the chemical scaffolds related to biological activity, we developed scoring functions as mathematical methods to predict the probability of new compounds to inhibit the bacterial collagenases, and further employed them to assess the suitability of repurposed non-antibiotic pharmacological agents as collagenase inhibitors.

## 2. Materials and Methods

### 2.1. Datasets Preparation

In order to determine the structural profile of bacterial collagenase inhibitors, we used the *Clostridium perfringens* (strain 13/Type A) collagenase as a model and enzyme coded CHEMBL2802 in the database of the European Institute of Bioinformatics (EMBL-EBI). The chemical and bioinformatics data resource (ChEMBL) was investigated to identify all compounds registered as inhibitors on the CHEMBL2802 target [43]. The obtained set of molecules was made up of the chemical structures of bacterial collagenase A (ColA) inhibitors (bacterial collagenase inhibitors set—BCI) and their corresponding Ki values (M). The Ki values were converted to their negative logarithmic values (pKi). All the structures were processed by removing charges and keeping the largest fragment.

DataWarrior 5.2.1. software [44] was used to calculate the compounds’ molecular weight (MW), logarithm of the partition coefficient (logP), number of hydrogen bonds donors (HBD), and number of hydrogen bonds acceptors (HBD). The intervals of distribution of these parameters were used to search the ChEMBL database. The resulting compounds were randomly chosen to prepare a decoy set (DCY) with 20 folds more entries than the BCI set. BCI and DCY sets were united into a single dataset, named ALL set.

The structures from the drug repository DrugBank 5.1.18 [45] were downloaded to search for new potential bacterial collagenase inhibitors. The inorganic and organometallic structures and the mixtures of compounds were removed from this set. The filters 250 ↔ 750 (MW), 7 ↔ 15 (HBA), 0 ↔ 6 (HBD), and −2.5 ↔ 6 (logP) were applied, resulting in a final database named DB.

### 2.2. Molecular Descriptors

An array of 1D and 2D molecular descriptors were calculated for all the structures in the ALL set using the freely available PaDEL-Descriptor v2.21 software [46], using the SMILES codes extracted from the ChEMBL as input. The zero variant variables were removed. The cutoff values for the relevant descriptors were established by using a receiver operating characteristic (ROC) curve analysis. The latter was performed using IBM SPSS Statistics v20.0 software (IBM SPSS Statistics for Windows, Version 20.0. Armonk, NY, USA: IBM Corp.). A flag variable (BC) was introduced to indicate if the value of the target descriptors is above the defined cutoff value and to indicate a potential BCI compound.

### 2.3. Bemis-Murcko Skeletons and Murcko Frameworks Analysis

Murcko frameworks (MF) and Bemis-Murcko (BM) skeletons, representing the structural molecular frameworks incorporating only the rings and their interconnecting chains, [47] were generated for both BCI and DCY sets by using DataWarrior. Two arrays of performance scores, noted as P-MF and P-BM, were calculated as the average value of the pKi values registered for all the compounds in set ALL sharing the sub-structure of interest (Equations (1) and (2)). The values of these scores are correlated with the odds for each sub-structure to generate active compounds towards the pharmaceutical target [48].

(1) $$P-{\mathrm{MF}}_{x}=\frac{1}{n}\sum_{1}^{n}\mathrm{pKi},\;\mathrm{if}\;{\mathrm{MF}}_{x}\;\mathrm{present}$$

(2) $$P-{\mathrm{BM}}_{x}=\frac{1}{m}\sum_{1}^{m}\mathrm{pKi},\;\mathrm{if}\;{\mathrm{BM}}_{x}\;\mathrm{present}$$

### 2.4. Plain Ring Analysis

DataWarrior 5.2.1 software was used to generate all ring systems existing in each compound from the ALL set. Each structure was divided in multiple fragments based on each cycle structure. The single bonded substituents were erased, while the double bonded heteroatoms connected directly to the ring system were kept. The P-PR performance score was calculated using Equation (3)—as the average value of the pKi values of all the compounds in set ALL sharing the respective plain ring (PR).

The odds variable (OD) was calculated for each PR fragment as the ratio of a PR fragment frequency in the BCI set compared to the DCY set (Equation (4)) and represents the likelihood of the scaffold to be associated with a BC inhibitor.

(3) $$P-{\mathrm{PR}}_{x}=\frac{1}{n}\sum_{1}^{n}\mathrm{pKi},\;\mathrm{if}\;{\mathrm{PR}}_{x}\;\mathrm{present}$$

(4) $${\mathrm{OD}}_{x}=\frac{\mathrm{counts}\;\mathrm{in}\;\mathrm{BCI}}{239}\times \frac{4780}{\mathrm{counts}\;\mathrm{in}\;\mathrm{DCY}}\;$$

### 2.5. Repurposing Study

The structures of the compounds in the DB set were transformed in BM and PR fragments. The S-PR score was defined as the average of the P-PR values of all PR fragments found in a compound and was used to estimate the probability of that compound to inhibit BC. A total performance score (TS) was used to take into account both the S-PR score and the P-BM values, as shown in Equation (5).

TS_i_ = P-BM_i_ + S-PR_i_ (5)

The repurposing candidates that exhibited high performance scores were thereafter screened against the ColA structure using molecular docking simulations.

### 2.6. Homology Modeling

Since there is no readily available crystal structure of *C. perfringens* collagenase A, we used homology modeling methods to build tridimensional models of the bacterial protease. The sequence of the target protein was retrieved from the UniProt database (code P43153) in FASTA format. SWISS-MODEL [49] web-server and YASARA Structure [50] software are both fully automated resources that were used to search for templates and to build the protein models. The resulting models were chosen for further studies considering the sequence similarity, coverage, presence of Zn^2+^ cofactor in the active site, and quality parameters. The quality assessment of generated models was performed with a SAVES v6.0 server, using ERRAT, VERIFY3D, PROVE, and PROCHECK programs [51,52,53,54].

The top ranked models were further validated by docking three highly potent ColA inhibitors into the active site using an induced fit approach, treating several key residues (e.g., Glu503, Glu534, Trp518, Tyr577, Tyr583) as flexible. The model that generated the most optimal conformations of the protein-ligand complexes was used for the virtual screening of the repurposing candidates.

### 2.7. Molecular Docking

A molecular docking experiment was employed for the virtual screening of the repurposable candidates to select hit molecules with potential ColA inhibitory activity. The docking studies were performed using the AutoDock Vina v1.1.2 [55] algorithm built within the YASARA Structure software. The docking grid box included only the active site within the peptidase domain, which is formed between two half-domains (central helix and gluzincin helix). The active site includes the catalytic Zn^2+^, which is complexed by two histidines (His502 and His506) and two histidine-stabilizing glutamates (Glu503 and Glu534).

The predicted protein structure that yielded the best results after induced fit docking of the selected strong inhibitors was used for further screening. The modelled protein and docking protocol were further validated by docking the BCI set into the active site and testing the correlation between experimental and predicted parameters that describe ligand efficiency.

Both protein structures and ligands were protonated according to the physiological pH (7.4). Three-dimensional structures of both BCI and DB sets were generated and energetically minimized with OpenBabel v2.4.1 software [56], using the GAFF force field (general AMBER force field) and 1500 steps with the steepest descent algorithm. The screening algorithm used rigid side chains and performed 12 docking runs for each ligand and the results were retrieved as the binding energy (ΔG, kcal/mol), ligand efficiency (ΔG\no. of heavy atoms), and dissociation constant (Kd, pM) of the best binding pose. The binding poses of chosen hit repurposable candidates were rescored using the AutoDock Vina local search algorithm and energy minimization with a NOVA forcefield. The analysis of predicted conformations of the protein-ligand complexes and molecular interactions was performed using the BIOVIA Discovery Studio Visualizer (BIOVIA, Discovery Studio Visualizer, Version 17.2.0, Dassault Systèmes, 2016, San Diego, CA, USA).

## 3. Results

### 3.1. Datasets

A dataset of 253 structures belonging to *Clostridium perfringens* collagenase inhibitors, was collected from the ChEMBL database together with their corresponding inhibitor constants (Ki, M). The dataset (BCI) was filtered by removing the imprecise biological values resulting in a final dataset that contains 239 compounds.

The compounds of the BCI set had MW values in the 302.3 ↔ 731.4 g/mol range, HBA between 8 and 14, HBD between 1 and 5, and the logP values situated in the −1.99 ↔ 5.58 interval. Based on these values, the limit points for establishing the decoy set (DCY) were: 250 ↔ 750 (MW), 7 ↔ 15 (HBA), 0 ↔ 6 (HBD), and −2.5 ↔ 6 (logP). The resulting 670,703 structures from ChEMBL were randomly selected to set up the DCY set. The DCY set consists of 4780 compounds, 20-fold more than the BCI set. Thus, the ALL set consists of 5019 structures).

From the 11,172 structures downloaded from DrugBank, after screening by employing the above-mentioned filters, 2685 structures were included in the final database.

### 3.2. Murcko Frameworks Profile

The Murcko framework (MF) consist of all the ring systems of each compound’s structure and the atoms uniting them. In a relationship with the original molecule, the MF fragments don’t contain any single bonded side-chain atoms. The BCI set compounds generated a number of 32 sub-structures (MF01-MF32), while the compounds in the DCY set yielded 4421 sub-structures. The null MF was produced only by compounds from the DCY set. The Shannon diversity index was calculated as 2.394 for the BCI set and 8.320 for the DCY set. This significant difference indicates that the nature of MF is important for the design of BC inhibitors.

**Figure 1:**
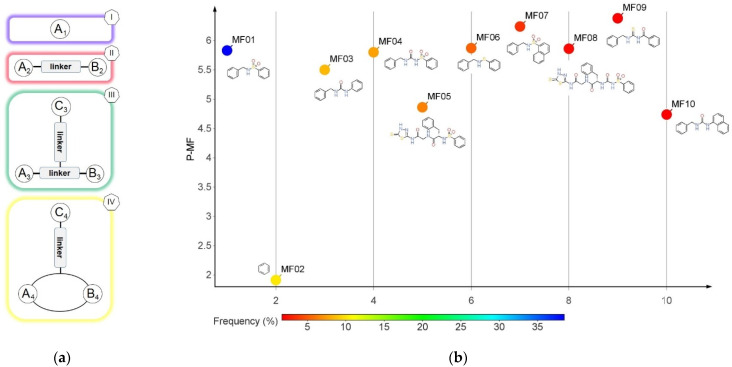
The Murcko framework structures: (**a**) The architecture types (I-IV) of the MF skeletons; (**b**) the performance score (P-MF) in relation with the frequency of distribution in the BCI set for MF01-MF10.

A number of 27 MF are strictly specific to the BCI set, while MF01, MF03, MF04, and MF10 are found almost exclusively in the inactive compounds set. The P-MF values ranged between 1.91 and 8.10. The structures and P-MF values for the most frequent frameworks observed in the BCI set are presented in Figure 1.

The MF frameworks can be classified in four major types based on their structure. The type I contains only one ring and is represented by the benzene (MF01) structure. The architectural type II is represented by two cyclic structures linked by a linear bridge of 4 ↔ 6 atoms. Type III is derived from type II, having an additional hexagonal ring (C3) attached to the linear linker. The skeletons in the type IV category are cyclic homologues of the type III template with 2 pentagonal rings inserted in a cycle of 15 up to 21 atoms. A hexagonal ring is bound by a one atom branch to the main linker, in a similar manner to the type III skeletons.

With the exception of MF02 (benzene ring), all MF sub-structures found in the set BCI contain at least two rings and have a P-MF value over four. As an MF sub-structure, the benzene ring is associated with a low potency of BC inhibition demonstrated by the P-MF value of 1.91.

The dimension of the side-chain elements attached to the MF core was quantified as the parameter SC. It was calculated by subtracting the MW of the MF sub-structure from the MW of the whole molecule. For the BCI set, the SC value ranged between 14.03 and 643.23 with an average of 170.01, while in the DCY set the distribution was from 0 (no side-chain elements) to 598.76 with an average of 115.6. The difference is statistically significant, indicating the importance of the presence of side-chain elements. The number of heavy atoms is also statistically different: in the BCI set, the average is 30.0 atoms, while in the DCY set, the average is 32.5.

### 3.3. Bemis-Murcko Skeletons Profile

Bemis-Murcko (BM) skeletons are derived from the molecules’ MF sub-structure by the removal of side-chain elements and of atom labels, and the changing of all bond types to single bonds. The 239 structures in the BCI set generated 21 distinct BM skeletons (BM01-BM21), while the 4789 compounds of the DCY set yielded 3068 BM structures. The null skeleton resulted from 0.46% of compounds in the set (22 structures). The Shannon diversity index values were calculated as 0.896 for the BCI set and 3.300 for the DCY set.

**Figure 2:**
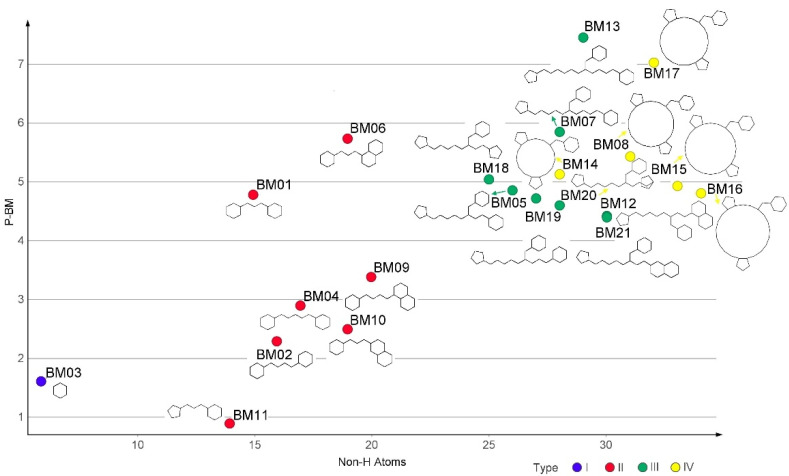
The structures of BM1-BM21 skeletons and the relation of their P-BM values depending on the number of carbon atoms and their types (I–IV).

The obtained P-BM values were in the range of 0.87–7.48 and seemed to increase with the number of non-H atoms in each BM scaffold. The four major architectural types of BM skeletons resulted from the BCI set, their structures, and the corresponding P-BM values are presented in Figure 2.

The type I of BM skeletons contains only the hexagonal ring of the BM3 structure and originates from the MF01 framework. The architectural type II is represented by 7 BM structures with P-MF values in the range of 0.87–5.75. Type III is derived from type II, while the BM skeletons in the type IV category are cyclic homologues of type III.

### 3.4. Plain Ring Analysis

The transformation of the 5019 structures from the ALL set in PR generated 1312 individual sub-structures, while the compounds from the BCI set generated only 17 PR fragments. All the ColA inhibitors from the BCI set contain at least one benzene ring (PR01). The second most frequent ring was the 1,3,4-thiadiazole-2-thione (PR02) with a frequency of 11.30%, followed by naphthalene (PR03) with a frequency of 7.95%, and thiophene (PR04) and quinolone (PR05), both found in 2.09% of the compounds from set BCI (Table 2). The pyrrolidine ring (PR06) was found in two BCI structures. The structure PR07-PR17 is found only in the BCI set compounds, and not in the DCY set.

The PR02 fragment registered the best P-PR value and the highest OD value, indicating its usefulness in designing new BC inhibitors. The naphthalene (PR03) and the benzene (PR01) presented OD values above one and fairly good P-PR values. Thiophene, quinolone, and pyrrolidine had OD values below one, and therefore a low predictable value in identifying new BC inhibitors.

**Figure 3:**
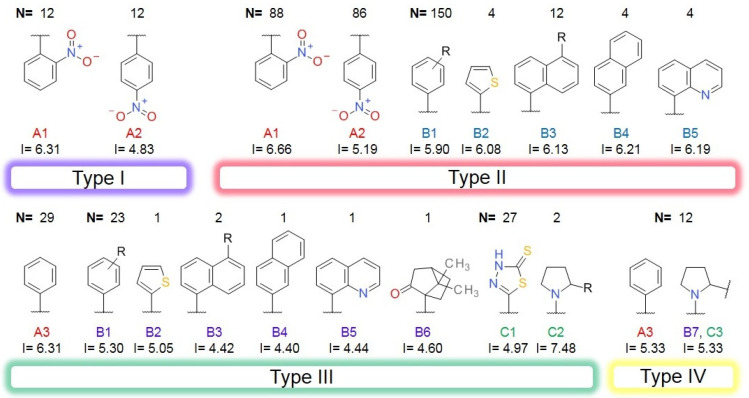
The plain rings (PR) as elements of each architectural type of BC inhibitor, together with their impact score (I) and number of occurrences (N) in the BCI set.

The PR analysis coupled with the BM architecture of the compounds from the BCI set indicated a high chemical similarity and highlighted the importance of some cyclic elements presented in Figure 3. The value I represents the average of the pKi values for each sub-structure across the BCI set.

The BCI compounds from types I and II contain ring A exclusively, a 2-nitrobenzene (A1) or a 4-nitrobenzene (A2) fragment. The value of I indicates a significantly higher potency for the 2-nitro substituted compounds compared to the 4-nitro derivatives. In types III and IV, the ring A is represented by a benzene (A3) with no other substituent. In the structure of types II and III inhibitors, the ring A is bound by a linker to the ring B, which is very similar in both types. The ring B1 represents a benzene with various substitution patterns, while rings B2, B4, and B5 are not substituted. The score I is significantly higher for the rings B2–B5 found in type II inhibitors, compared to the type III structures, indicating the importance of the overall geometry.

Most of the type III compounds have, as a C ring, the 1,3,4-thiadiazole-2-thione (PR-02), while only 2 compounds contain a 2-substituted pyrrolidine ring (PR06). The 12 compounds sharing the type IV structural architecture have the same ring elements A3, B7, and C3, with the 2 pyrrolidine cycles bounded in a large cyclic structure that has branched a benzyl fragment. The differences in the potency of these compounds comes from the dimension and the elements of the cyclic structure linking rings B and C.

### 3.5. Classification Model

All the structures form the ALL set were introduced in the PaDEL software and a number of 1444 1D and 2D descriptors were computed. After removing those with 0 variance, 1192 descriptors remained. A ROC analysis was executed for them, returning 8 descriptors with values of the area under the curve (AUC) parameter over 0.9. A cutoff value was established for each descriptor, considering 100% sensitivity and the highest possible specificity (Table 3).

The analysis of the descriptors and their cutoff values indicated the use of MDEO-11, AATS0s, ASP-0, and MAXDN as the best way to filter potential BCI compounds. The flag value BC was calculated using the following equations:

BC = 1, IF MDEO-11 > 1.194 AND AATS0s > 5.239 AND ASP-0 > 0.714 AND MAXDN > 2.490(6)

BC = 0, IF MDEO-11 ≤ 1.194 OR AATS0s ≤ 5.239 OR ASP-0 ≤ 0.714 OR MAXDN ≤ 2.490(7)

where, when the BC variable takes the value 1, it indicates a BCI compound. This method of identification has a sensitivity of 100% and a specificity of 89.58%.

The MDEO-11 descriptor is based on the molecular graph and accounts only for oxygen atoms bonded to only one non-hydrogen atom, irrespective of bond types (primary oxygen atoms) [57]. The MDEO-11 value represents the number of primary oxygen atoms divided by the geometrical average of the graph distances between each pair of primary oxygen atoms. In the BCI set, this type of oxygen atom can be found in groups like: -OH, >C=O, -NO_2_, and -SO_2_-. For a compound to have a value above the cutoff of 1.194, it needs at least 3 such oxygen atoms. The distance between these atoms is also very important, as the descriptor value decreases as the distance increases. Considering type I inhibitors, they need at least an oxygen atom in the chain close to the nitro group.

The ASP-0 descriptor represents the SP-0 descriptor divided by the number of atoms in the compound’s structure. SP-0 is based on the molecular graph and takes account of the number of edges for each node. It is calculated as a sum of the reciprocal square root for each number of edges in a node. In the class of n-alkanes, the highest possible value of ASP-0 is 1, found in the case of methane and ethane, and decreases as the carbon chain is longer. The branched derivatives have higher ASP-0 values compared to their unbranched isomers.

Both AATS0s and MAXDN use the Kier–Hall intrinsic state values (Is). AATS0s is calculated as the sum of each atom squared Is value, divided then by the number of all atoms. For a compound to have an AATS0s value above 5.239, it needs atoms like fluorine, chlorine, oxygen, nitrogen, or multiple bonded carbon atoms. The MAXDN represents the maximum negative value registered in a compound for the sum of the ratio of differences of Is values and the corresponding topological distances. It is considered a measure of the molecule’s nucleophilicity [58].

### 3.6. Homology Modeling and Molecular Docking of ColA Inhibitors

A molecular docking study was performed for ColA inhibitors to validate a virtual screening protocol for repurposing DB candidates. The 3D structure of *C. perfringens* ColA has not been solved yet; therefore, several models of the protein were built using two structure prediction tools. The first four templates identified by SWISS-MODEL and YASARA ranked by sequence coverage were used for model generation. The quality parameters of the built models are shown in Table 4. Both tools found crystal structures 2Y3U, 4ARE, and 4AR9 as potential templates, while 5O7E was identified only by SWISS-MODEL, and 5IKU by YASARA, respectively. The best overall quality factor calculated with ERRAT was found for the model built using 2Y3U with YASARA. However, this structure lacks the catalytic Zn^2+^ and could not be used for further studies. On the other hand, the worst overall quality factor was calculated for the model generated using 5IKU structure (76.9874), which has only 21% coverage of the ColA sequence and shares only 35.37% sequence identity. The structure built using the 5IKU template corresponds to the ColA collagen-binding domain and is not suitable for the aim of this study. The models generated using 2Y3U and 4ARE (69 and 60% coverage, 49.27 and 49.78% sequence identity) crystal structures included the metalloprotease S1 domain, consisting of an activator domain, a catalytic subdomain, and a helper subdomain. Models built using 4AR9 and 5O7E included only the catalytic and helper subdomains (35% coverage, 55.55%, and 58.44% sequence identity), which were sufficient for the molecular docking simulations.

**Figure 4:**
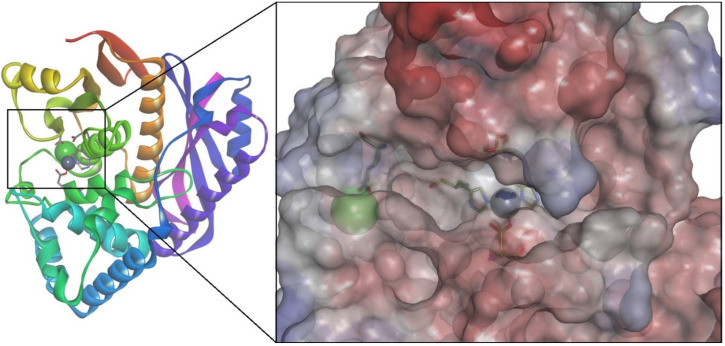
Predicted 3D structure of collagenase A catalytic and helper domains from *C. perfringens* (model S4). The surface of protease active site is colored by pKa values. Ca^2+^ is depicted as a green sphere, Zn^2+^ as a purple sphere, and metal-binding residues are highlighted.

Three potent ColA inhibitors from different chemical classes were docked into the catalytic site using an induced fit approach to select the most suitable target protein for further screening. The molecular docking experiment was performed for models Y1, Y3, S4, and Y5 using an oxime derivative (CHEMBL306726, pKi = 8.3 M), a 1,3,4-thiadiazole-2-thione derivative (CHEMBL3142607, pKi = 6.7), and a peptide analogue inhibitor with a phosphonyl group (CHEMBL2115242, pKi = 8.1). Only docking with model S4 (Figure 4) yielded satisfying results regarding the binding poses of all three inhibitors.

**Figure 5:**
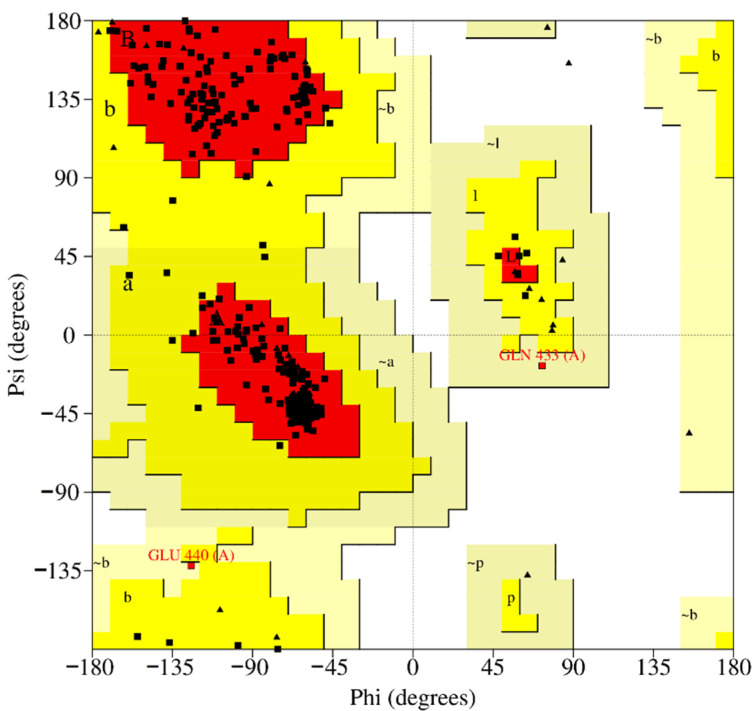
Amino acid distribution in the Ramachandran plot for model S4. The phi-psi torsion angle for two residues (0.6%) are in generously allowed regions (~a, ~b, ~l, ~p) and are highlighted in red.

This model had a 95.0954 overall quality factor. In general, proteins with high resolution structures have overall quality factors around 95 or higher. The Z-score of the model was 0.291. Moreover, the analysis of the Ramachandran plot showed that the phi-psi torsion angle for 92.5% of residues are in the most favored regions, 23 residues (6.9%) are in additional allowed regions, 2 residues (Gln433 and Glu440) are in generously allowed regions, while 0 residues fall into disallowed regions (Figure 5).

**Figure 6:**
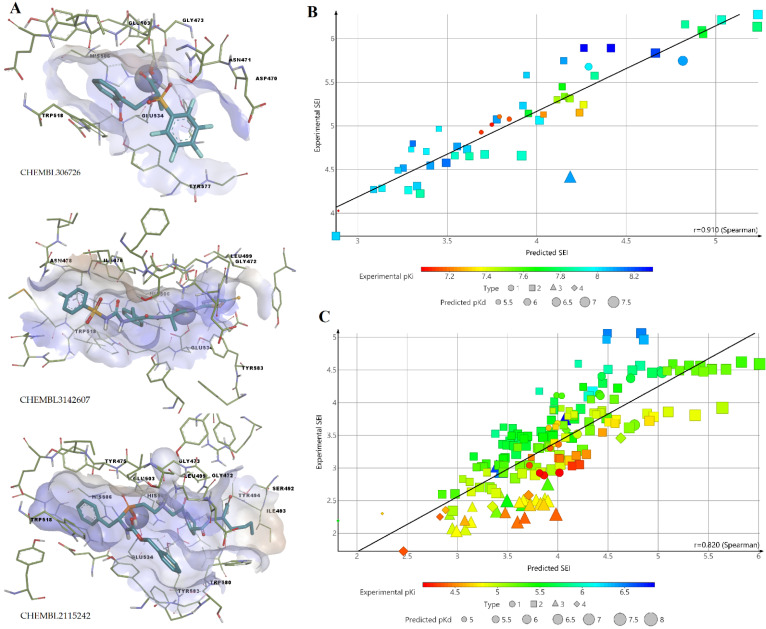
Docking analysis results: (**A**) Binding poses of three selected inhibitors for ColA model validation; (**B**) correlation diagram between experimental and predicted SEI values for compounds from BCI set with strong activity; (**C**) correlation diagram between experimental and predicted SEI values for compounds from BCI set with weak activity.

The binding poses of the three inhibitors in the active site are shown in Figure 6A. All three inhibitors interact with the catalytic zinc. The docking protocol was further validated by docking the BCI set and calculating the determination coefficient between experimental and predicted values of ligand efficiencies. Good correlations were obtained between experimental and predicted surface-binding efficiency index values (SEI), after splitting the BCI set into strong (pKi > 7, R^2^ = 0.8281) and weak (pKi < 7, R^2^ = 0.6724) inhibitors (Figure 6B,C). SEI is calculated as the activity value divided by polar surface area/100 Å).

The docking scores (ΔG) of the compounds from the BCI set ranged from −11.14 to −6.23 kcal/mol with a mean value of −8.35 kcal/mol. Forty-seven amino acids are involved in overall interactions with the compounds from the BCI set, the most frequent being Asn471 (100%), Tyr583 (95.78%), Gly473 (75.53%), Glu534 (68.78%), and Ile474 (67.93%). On average a compound from the BCI set interacts with of 15.63 amino acid residues (values ranging between 11 and 26).

### 3.7. Repurposing Study

The compounds from the DB set were transformed in their corresponding BM and PR fragments. A total of 194 structures were found to generate one of the active types of BM structures (BM01–BM21). Among these molecules, 24 are classified as approved, 137 as experimental, and 40 as investigational drugs. A total number of 112 compounds share the BM03 sub-structure, belonging to type I architecture, while 82 compounds have a type II BM sub-structure.

The DB set was searched to find the compounds that generate one of the PR01–PR17 structures, thus identifying 1806 compounds. For each compound the average of the P-PR values (S-PR) was calculated to estimate the probability to inhibit BC. A number of 1806 compounds produced at least one of the target PR fragments and their calculated S-PR value ranged from 0.065 to 2.669.

The DB compounds were used to calculate the BC flag value based on the chemical descriptors computed by PaDEL software. The value 1 that indicates a potential BC inhibitor was obtained for 664 compounds.

The sum of S-PR and P-BM values were added to obtain the TS score to select the DB compounds with the best chances to inhibit ColA. Of all the 2685 compounds of the DB set, only 194 presented TS values over 1, and 100 of them also had BC values equal to 1. These compounds were chosen as candidates for molecular docking screening. The compounds with the best 10 TS values are presented in Table 5 along with their main target of action as registered in DrugBank.

The compound DB08498 is the chloro-derivative of DB08497, both compounds being under development as caspase-3 inhibitors. DB03124 and DB08229 are also caspase-3 inhibitors. Caspase-3 is a cysteine protease that cleaves several proteins after aspartic acid residues in specific sequences [59]. The results indicate a possible similarity between the structures of the catalytic domain of both enzymes. The compound DB07556 targets both the macrophage metalloelastase and the interstitial collagenase, metalloproteases that are similar with ColA. DB07290 inhibits the *Bacillus anthracis* lethal toxin, a zinc-dependent peptidase presenting the consensus sequence HEXXH [60], similar with ColA.

The binding energy, ligand efficiency, and dissociation constant obtained from docking simulations for compounds with the best TS values are shown in Table 6.

Of the 100 candidates, only 10 are approved for human use: benzthiazide, bortezomib, ioxitalamic acid, entacapone, technetium tc-99 m disofenin, chloramphenicol succinate, mebrofenin, lodoxamide, betiatide, and nafcillin. The ioxitalamic acid, technetium tc-99 m disofenin, mebrofenin, and betiatide are all used for diagnostic purposes and have a low druglike character because of their toxicity risks [61,62]. Chloramphenicol succinate and nafcillin are antimicrobial drugs [63] and their effects on bacterial growth can promote resistant strains limiting their development as BCI. Benzthiazide (thiazide diuretic), entacapone (catechol-O-methyltransferase inhibitor, Parkinson’s disease) and lodoxamide (mast cell stabilizer, antiallergic) emerged as the best candidates for development as anti-virulence agents in infections with pathogens form the *Clostridium*, *Pseudomonas*, *Vibrio*, and *Streptomyces* genuses.

The predicted binding affinities, ligand efficiencies, and the residues involved in H-bonding are presented in Table 7. All three hit compounds showed ligand efficiency values above 0.35, which indicates good binding to the target protein. Among these molecules, benzthiazide exhibited the highest binding affinity, with −9.54 kcal/mol binding energy and 6.99 M pKd. Entacapone and lodoxamide had lower affinities and pKd values, correlated with the smaller TS score.

**Figure 7:**
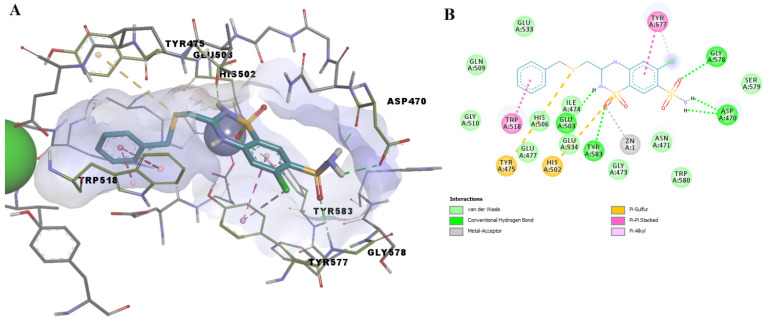
(**A**) 3D binding pose of benzthiazide into ColA active site; (**B**) 2D molecular interactions diagram for benzthiazide-ColA complex.

The molecular docking simulations showed that benzthiazide forms a direct metal-acceptor interaction with the catalytic zinc via the sulfone moiety within the thiazide scaffold (Figure 7). It forms five hydrogen bonds with Gly578, Tyr583, Asp470 and the catalytic Glu503. The protein-ligand complex is further stabilized by forming several nonpolar interactions, such as pi-sulfur, pi-pi stacked, pi-alkyl and van der Waals interactions.

**Figure 8:**
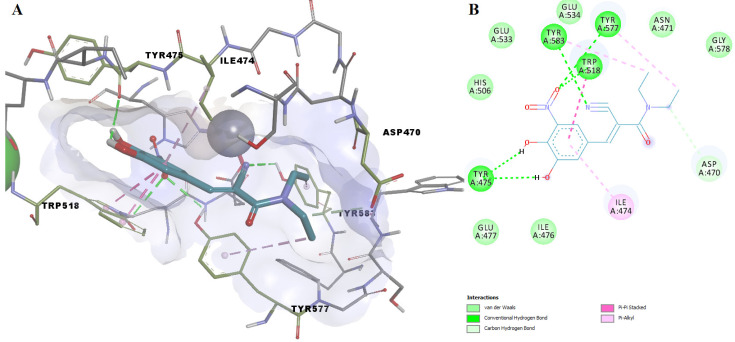
(**A**) 3D binding pose of entacapone into ColA active site; (**B**) 2D molecular interactions diagram for entacapone-ColA complex.

In the docking experiment, entacapone binds into the ColA active site via five hydrogen bonds with four residues (Tyr475, Trp518, Tyr577, Tyr583). A carbon-hydrogen bond, pi-alkyl, and pi-pi stacked interactions are also present (Figure 8). Although entacapone does not interact directly with Zn^2+^, it interacts with metal-binding residues Glu533, Glu534, and His506 through van der Waals weak forces. Moreover, the substituted nitrobenzene moiety interacts with the binding pocket in a similar fashion with the docked ColA inhibitors from the BCI set (Figure 6A).

**Figure 9:**
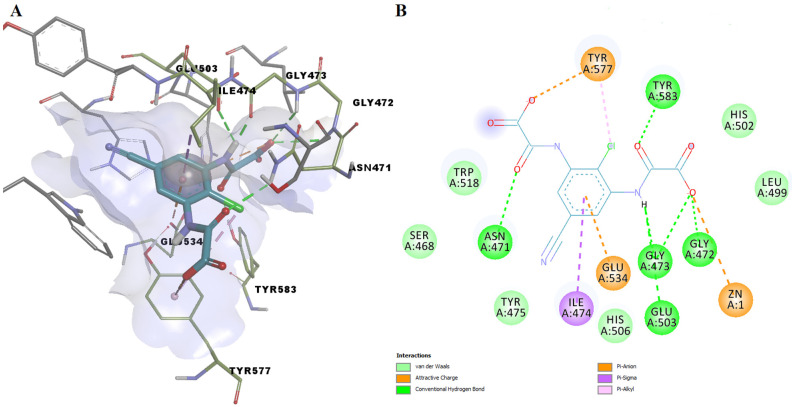
(**A**) 3D binding pose of lodoxamide into ColA active site; (**B**) 2D molecular interactions diagram for lodoxamide-ColA complex.

The predicted binding pose of lodoxamide into the ColA active site showed an attractive interaction between the negatively charged carboxyl moiety and the catalytic zinc. Lodoxamide binds to the enzyme via six hydrogen bonds formed with five residues (Asn471, Gly472, Gly473, Tyr583 and catalytic Glu503). Moreover, several nonpolar interactions are formed with the binding pocket, such as van der Waals, pi-sulfur, pi-sigma, and pi-lakyl interactions (Figure 9).

## 4. Discussion

The results of the structural analysis indicated the importance of the molecular geometry for the biological activity, as a limited array of scaffolds and cyclic structures were found to be connected with the capacity to inhibit ColA. The descriptors observed as relevant in discriminating active and inactive compounds are based also on the molecular geometry, highlighting its importance. The presence of certain types of atoms or groups of atoms seems to be essential as well. A quantitative structure-activity relationship (QSAR) study on 5-amino-2-mercapto-1,3,4-thiadiazole derivatives that inhibit the related *Clostridium histolyticum* collagenase indicated the importance of an amide function close to a sulfonamide group and the branching of the molecule [64]. Our research confirms these results and generalizes them using the number of primary oxygen atoms and their relative distances.

The use of scoring methods coupled with the selected molecular descriptors proved to be useful in identifying several metalloproteases inhibitors. The method was initially implemented to prioritize the compounds in the docking studies but proved to render good results on its own. It can be easily used for any database of compounds but needs their transformation to their corresponding Bemis-Murcko skeletons, plain rings, and molecular descriptors. The identified architectural types are a simple method to prepare analogs of the obtained candidates in the process of lead optimization.

A total of 100 repurposable candidates were subjected to molecular docking simulations to identify potential ColA inhibitors. The docking results revealed that benzthiazide and lodoxamide can interact directly with the Zn cofactor, while entacapone interacted with the amino acid residues within the active site. These results are similar with the pharmacological profile of benzthiazide. Contrary to other thiazide class compounds, it binds strongly to all human carbonic anhydrases isoforms coordinating the zinc ion through the sulfonamide group [65].

The major limitation of the study is the small number of known inhibitors and their low structural diversity. The chances of finding new lead compounds are hindered by these problems and the methods we used prioritized sensitivity over specificity as a mean to overcome this drawback. This approach may have yielded a higher number of false positives. The candidates need to be confirmed by in intro assays to be further developed as repurposed agents.

## 5. Conclusions

A virtual screening algorithm was implemented using both ligand-based and structure-based drug discovery approaches to identify novel potential inhibitors of *C. perfringens* collagenase A. Benzthiazide, entacapone, and lodoxamide are three approved drugs that we propose as potential repurposable anti-virulence agents, based on the predicted probability of inhibiting the bacterial collagenase and the favorable simulated interactions with the enzyme. Further studies are needed for the selected molecules to confirm the predicted biological activity and to assess the efficacy in treating *C. perfringens* infections.

## Figures and Tables

**Table 1:** Collagenases associate with pathogenic bacteria.

Pathogen	Disease	Collagenase	MEROPS ID	Class	Substrate	Reference
*Clostridium histolyticum*	Clostridial myonecrosis	Collagenase H	M09.003	Class II	Type I, II, and III collagens	[20,21]
		Collagenase G/A	M09.002	Class I	Type I, II, and III collagens	
*Clostridium tetani*	Tetanus	Collagenase col T	M09.005			[22]
*Clostridium perfringens*	Clostridial myonecrosis	Collagenase G/A (or collagenase A g.p.)	M09.002	Class I	Type I collagen, Pz peptide, azocoll	[23]
*Vibrio alginolyticus*, *Vibrio parahaemolyticus*, *Vibrio vulnificus*	Cellulitis, septicemia	Collagenase V	M09.001	Class III collagenases	Gelatin, casein, collagen, synthetic substrate	[24,25]
*Vibrio mimicus, Vibrio parahaemolyticus*, *Vibrio cholerae*	Gastroenteritis	VMC peptidase	M09.004	Class II collagenases	Type I, II, III collagens, gelatin, Cbz-GPLGP, Cbz-GPGGPA	[14,24,25]
*B. cereus*	Periodontal disease, endophthalmitis	Collagenases Q1	M09.002/M09.003	Class I collagenases/ class II	Type I, II, III collagens	[26,27]
*Porphyromonas gingivalis*	Periodontal disease	Collagenase (Porphyromonas type)	U32.001	Not applicable	Soluble and reconstituted fibrillar or heat-denatured type I collagen,	[28]
*Helicobacter pylori*	Gastro-duodenal ulcer	Collagenase (Helicobacter type)	U32.002	Not characterized	Not characterized	[29]
*Aeromonas veronii*	arthritis, gastroenteritis, meningitis, septicemia	Collagenase (Salmonella type)	U32.003	Not characterized	Not characterized	[30,31]
*Salmonella* sp.	Salmonellosis (associated with abomasitis, peritonitis and polyserositis)					
*Escherichia Coli*	Urinary infections, digestive infections, etc.	YhbV	U32.A.01	Not characterized	Not characterized	[17]

**Table 2:** The structures of the most frequent PR fragments generated in the BCI set and their P-PR scores and odds values.

Code	PR01	PR02	PR03	PR04	PR05	PR06
Structure	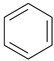	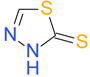	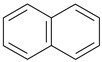	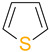	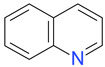	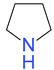
P-PR	0.368	4.971	1.063	0.281	0.291	0.065
Frequency (%) BCI	100.00	11.30	7.95	2.09	2.09	0.84
Frequency (%) DCY	78.47	0.56	2.20	2.18	2.11	4.79
OD (BCI vs. DCY)	1.27	20.00	3.62	0.96	0.99	0.17

**Table 3:** List of molecular descriptors capable of identifying the BCI compounds against the DCY structures and their cutoff value for 100% sensitivity.

Code	Type	Cutoff	Descriptor’s Mathematical Representation
MDEO-11	MDE	>1.194	Molecular distance edge between all primary oxygens
AATS5s	Autocorrelation	>3.315	Average Broto–Moreau autocorrelation—lag 5, weighted by I-state
AATSC0s	Autocorrelation	>2.008	Average centered Broto–Moreau autocorrelation—lag 0, weighted by I-state
AATS0s	Autocorrelation	>5.239	Average Broto–Moreau autocorrelation—lag 0/weighted by I-state
ASP-0	ChiPath	>0.714	Average simple path, order 0
maxHBint5	Electrotopological State Atom Type	-	Maximum E-State descriptors of strength for potential Hydrogen Bonds of path length 5
meanI	Electrotopological State Atom Type	>2.370	Mean intrinsic state values I
MAXDN	Electrotopological State Atom Type	>2.490	Maximum negative intrinsic state difference in the molecule

**Table 4:** Quality parameters of the generated models of *C. perfringens* ColA.

Method	Model	Template	ERRAT (Overall Quality Factor)	VERIFY3D (3D-1D Score >0.2% Residues)	PROVE (Buried Outlier Protein Atoms Total, %)	Residues in Most Favored Regions (%)	Residues in Disallowed Regions (%)
SWISS-MODEL	S1	2Y3U	96.7016	97.93	4.7	93.2	0.2
	S2	4ARE	97.1299	98.51	4.2	92.9	0.5
	S3	4AR9	96.5517	96.12	4.1	91.9	0.0
	S4	5O7E	95.0954	92.80	4.6	92.5	0.0
YASARA	Y1	4ARE	98.3824	98.41	3.1	93.4	0.3
	Y2	2Y3U	99.1098	97.41	3.3	94.2	0.3
	Y3	4AR9	98.7310	99.02	4.5	92.6	0.0
	Y4	5IKU	76.9874	90.44	3.5	86.9	0.0
	Y5	hybrid	97.3529	99.42	2.5	93.6	0.5

2Y3U—collagenase G (ColG) from *Clostridium histolyticum*; 4ARE—ColG from *C. histolyticum*; 4AR9—collagenase T (ColT) from *C. tetani*; 5O7E—collagenase H (ColH) from *C. histolyticum*; 5IKU—ColG from *C. histolyticum.*

**Table 5:** The top DrugBank candidates based on their TS value and their known pharmacological targets.

Code	Name	TS	Target
DB08498	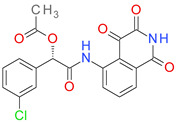	6.11	• Caspase-3
DB08497	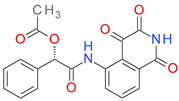	6.11	• Caspase-3
DB01689	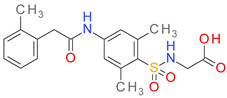	5.15	• Aldose reductase
DB07030	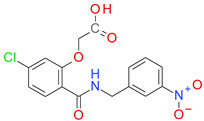	5.15	• Aldose reductase
DB07556	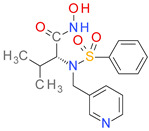	5.15	• Macrophage metalloelastase • Interstitial collagenase
DB06989	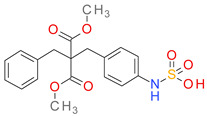	5.15	• Receptor-type tyrosine-protein phosphatase beta
DB07290	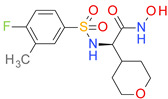	5.15	• Anthrax lethal factor endopeptidase
DB03124	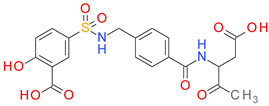	5.15	• Caspase-3
DB04659	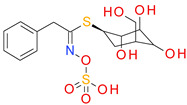	5.15	• Lactase-phlorizin hydrolase
DB08229	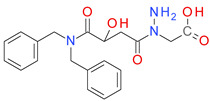	5.15	• Caspase-3

**Table 6:** Molecular docking simulations results for the top ranking DrugBank candidates.

Code	LE (kcal/mol Per Heavy Atom)	ΔG (kcal/mol)	pKd (M)	No. of Contacts
DB08498	0.3483	−9.751	7.148	18
DB08497	0.3476	−9.386	6.880	18
DB01689	0.3545	−9.572	7.016	18
DB07030	0.3472	−8.68	6.363	17
DB07556	0.2664	−7.192	5.272	16
DB06989	0.2938	−8.226	6.030	15
DB07290	0.3643	−8.379	6.142	16
DB03124	0.3114	−9.965	7.304	21
DB04659	0.3082	−8.013	5.874	16
DB08229	0.3352	−9.386	6.880	19

LE—ligand efficiency; ΔG—binding energy; pKd—negative logarithmic value of predicted dissociation constant.

**Table 7:** Molecular docking simulations results and TS performance score for the best repurposing candidates.

Name	Code	TS	LE (kcal/mol Per Heavy Atom)	ΔG (kcal/mol)	pKd (M)	H-Bonding Residues	H-Bond Length (Å)
Benzthiazide	DB00562	2.85	0.3669	−9.539	6.992	Gly578	2.155
						Tyr583	2.246
						Asp470	1.839
						Asp470	2.328
						Glu503	2.095
Entacapone	DB00494	1.96	0.3521	−7.746	5.678	Trp518	2.987
						Tyr577	2.891
						Tyr583	2.690
						Tyr475	1.908
						Tyr475	2.592
Lodoxamide	DB06794	1.96	0.3744	−7.862	5.763	Asn471	2.647
						Gly472	2.720
						Gly473	2.844
						Gly473	1.732
						Tyr583	1.753
						Glu503	2.870

## Data Availability

Publicly available datasets were analyzed in this study. This data can be found here: https://www.ebi.ac.uk/chembl/ and https://go.drugbank.com/. Access date: 6 September 2021.
